# Surgical treatment of asymptomatic epithelioid hemangioendothelioma originating from the superior vena cava: A case report

**DOI:** 10.1097/MD.0000000000019859

**Published:** 2020-04-17

**Authors:** Seok Soo Lee, Jang Hoon Lee

**Affiliations:** a Department of Thoracic and Cardiovascular Surgery, Yeungnam University Medical Center; b Department of Thoracic and Cardiovascular Surgery, Yeungnam University college of Medicine, Daegu, Republic of Korea.

**Keywords:** epithelioid, hemangioendothelioma, thoracic surgery, vascular neoplasm, vascular surgical procedures

## Abstract

**Rationale:**

Epithelioid hemangioendothelioma is a rare endothelial tumor with a low-grade malignancy. This tumor can be treated with complete resection.

**Patient concerns:**

A 20-year-old Korean man visited our hospital due to an abnormal finding on standing chest PA X-ray. He did not have any past medical history.

**Diagnosis:**

Chest computed tomography shows a well-defined, oval-shaped tumor invading the brachiocephalic vein and superior vena cava. A malignant tumor of vascular origin was diagnosed by a percutaneous needle biopsy.

**Interventions:**

We performed en-bloc resection including the great vessels for complete resection of the tumor. Histologic evaluation confirmed the lesion to be a hemangioendothelioma and the surgical margins were free from tumor invasion.

**Outcomes:**

Fourteen days later, the patient was discharged without any complication. Thirty months after surgery, recurrences, or metastasis were not detected.

**Lessons:**

Epithelioid hemangioendothelioma is a rare malignant endothelial tumor in the central vein. Surgery is the treatment of choice and shows good results. We introduce and appropriate surgical method to ensure successful treatment for rare disease.

## 1 Introduction

Epithelioid hemangioendothelioma (EHE) is a rarely reported malignant tumor of vascular origin.^[1]^ It is found most often in the lung and liver, but rarely involves large veins such as innominate vein or superior vena cava (SVC).^[1,2]^ This tumor is a low-grade malignant neoplasm and can be treated with complete resection; although available, adjuvant treatment is still controversial. We describe here the successful surgical treatment of EHE arising from the intrathoracic great vessel.

## 2 Case report

**Figure 1:**
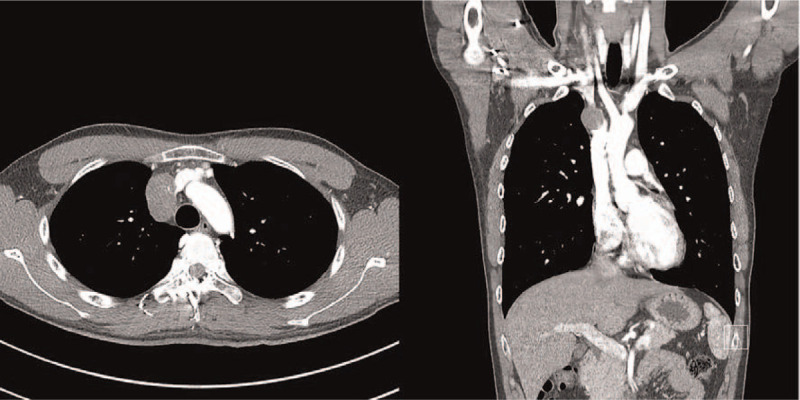
Computed tomography shows a 3.8 × 2.5 cm sized, well-defined mass with smooth margins, tiny punctate calcifications and mild contrast enhancement of the intrathoracic great vessel.

**Figure 2:**
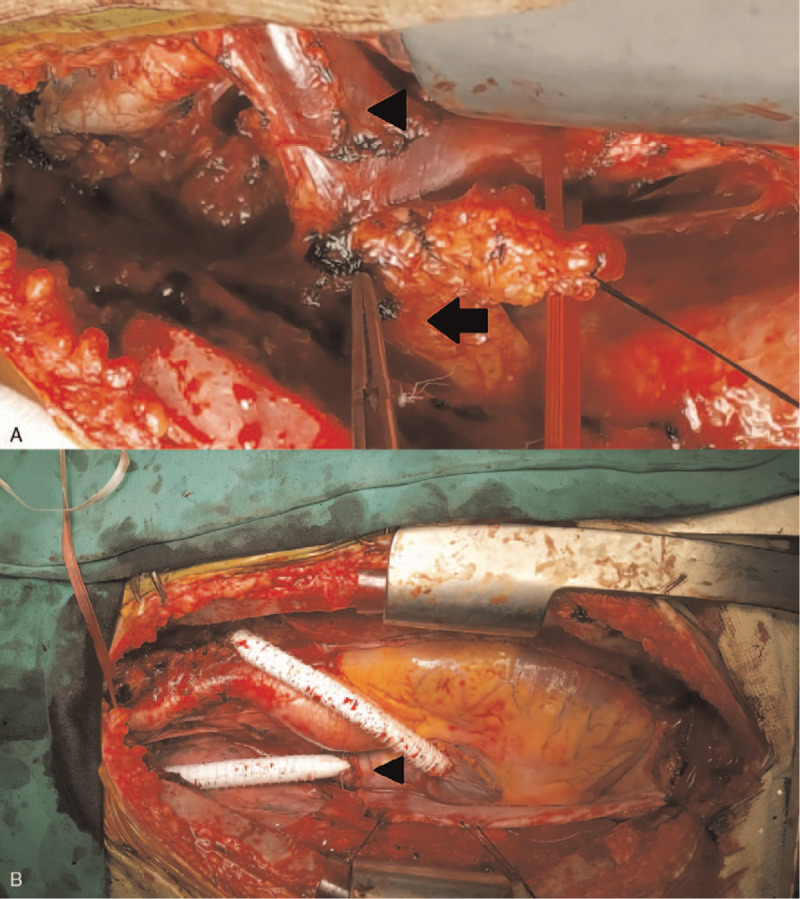
Perioperative view of the tumor: (A) Invasive mass obstructing the superior vena cava, “arrow head” indicates innominate vein, “arrow” indicates tumor. (B) Final view of reconstruction of the superior vena cava and innominate vein using a 10 mm Gore-tex removable ring, “arrow head” indicates superior vena cava.

**Figure 3:**
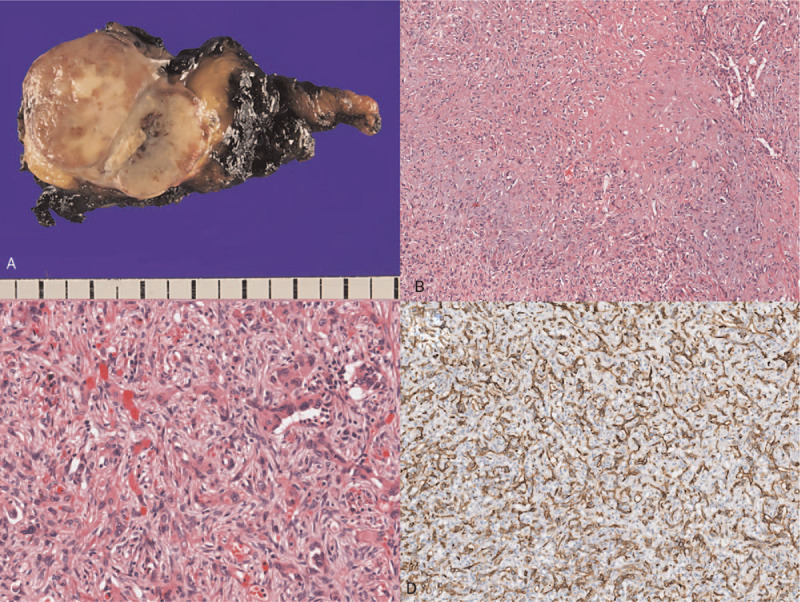
The mass measured about 3.7 cm. (A) The cut surface showed a tan to white color, with internal hemorrhage and calcification. Although the mass was relatively well-circumscribed, infiltration into the surrounding adipose tissue was also identified. (B) Center of the tumor was relatively hypocellular and had myxohyaline stroma. (C) Periphery of the tumor was hypercellular with abundant epithelioid tumor cells forming irregular vasculature or pseudoglandular pattern. The tumor cells showed marked pleomorphism, and some tumor cells had an intracytoplasmic lumen containing RBCs. (D) The tumor revealed positive staining for CD31.

A 20-year-old Korean male patient was referred to our hospital after an abnormal finding was detected in a simple chest scan during a military physical examination. He was subjected to a contrast-enhanced chest computed tomography scan. A 3.8 × 2.5 cm sized, well-defined, oval-shaped tumor invading the brachiocephalic vein and superior vena cava was found, showing homogeneous enhancement with calcifications (Fig. 1). Although the brachiocephalic vein was almost obstructed, the patient remained asymptomatic. To determine the course of treatment, identification of the tumor type was necessary. A percutaneous needle biopsy was conducted, and a malignant tumor of vascular origin was diagnosed. Since this tumor was not a good candidate for chemotherapy or radiation, we planned a surgical resection. The surgery was performed through a median sternotomy by dissecting the thymic tissue and approaching the tumor. Some parts of the mediastinal pleura found within the tumor were also removed. The tumor had a clear boundary with the aorta and was well demarcated. However, the mass had intruded into the innominate vein, brachiocephalic vein and SVC. For complete resection of the tumor, we determined an en bloc resection involving the great vessels. To ensure that blood flow was maintained through the brachiocephalic vein to SVC, an innominate vein was clamped using a vascular clamp at the distal end and was cut off to ensure a sufficient resection margin, which was then subsequently connected from the innominate vein to the right atrium using a 10 mm Gore-tex removable ring. The venous drain was sufficient after the bypass from the innominate vein to right atrium, and hence use of a cardiopulmonary bypass machine was not required during the surgery. An azygos vein was double ligated and divided. The SVC was clamped and cut between the tumor and right atrium with sufficient distance. The brachiocephalic vein was clamped and cut with a good distance. Mass was extensively removed along with surrounding vessels and tissues. A phrenic nerve was cut off as SVC was involved in the en bloc resection. End-to-end anastomosis of the brachiocephalic vein and SVC was performed using a 10 mm Gore-tex removable ring (Fig. 2). A diaphragm plication was performed because of a phrenic nerve injury. Since facial edema was noted, ventilator care was applied at the intensive care unit for 1 day. The patient was discharged from the hospital without any postoperative complications. Histopathology of the tumor mass revealed that the surgical margins were free from tumor invasion. It was pathologically determined that the tumor originated from the great vessels. Immunohistochemical staining for CD31, ERG, and CAMTA1 revealed diffuse positive, weakly positive and diffuse nuclear positive, respectively (Fig. 3). Based on the results of the pathological findings, our professors diagnosed the tumor as an EHE. After 30 months of surgery, the patient came for a follow-up chest computed tomography. No recurrences or metastasis were detected, and the flow of the artificial blood vessels was well-maintained. Currently, the patient continues taking anticoagulants. The patient provided written informed consent for publication of clinical details and images.

## 3 Discussion

The first EHE defined by Weiss and Enzinger in 1982 was a low-grade or borderline malignant lesion similar to angiosarcomas due to its local aggressiveness and metastasizing potential.^[1,2]^ This tumor was composed of epithelioid endothelial cells, characterized by their round or polygonal shape, abundant eosinophilic hyaline cytoplasm and the presence of cytoplasmic vacuoles and vesicular nuclei.^[1]^ EHEs are derived from endothelial cells and can occur in any part of the body, including the skin, liver, lung, or the extremities.^[3]^ EHE is a rare malignant tumor of vascular origin, and rarely develops in the large veins; most are hepatic EHE that spread to the inferior vena cava.^[4]^ Within the thorax, pleural, and pulmonary EHEs are more common than mediastinal EHE.^[5]^ Extracardiac mediastinal EHE, such as the innominate vein, azygos vein, and the SVC, are uncommon.^[2]^ In our case, the tumor was found to originate from the intrathoracic great vessel (brachiocephalic vein and SVC).

Patients are often asymptomatic, but edema or thrombophlebitis can develop.^[1,4]^ A diagnosis is often incidentally discovered after abnormal chest radiologic findings.^[1]^ Complete en bloc resection is the treatment of choice to control all the major thoracic veins.^[3]^ In our case, we also performed wide resection and had a clear resection margin. The use of cardiopulmonary bypass was not required since the tumor was remote from the heart, and venous drains from the head were maintained. The use of a ringed PTFE prosthesis has been widely described for the reconstruction of SVC,^[1]^ which prove beneficial since surgical therapy is the only treatment option for EHE. The role of adjuvant treatment remains unclear because of poor efficacy.^[2,6]^ A definitive diagnosis of EHE must be based on postoperative pathological findings. Classical histological findings include nests and cords of epithelioid endothelial cells distributed in the myxohyaline stroma, and evidence of intracytoplasmic lumina.^[4,5]^ In the current case, we found that the tumor center was relatively hypocellular and revealed myxohyaline stroma. Immunohistochemically, tumors are often positive for at least 1 endothelial marker (factor VIII-related antigen, CD31, CD34)^[5,7]^; our tumor tested positive for CD31 immunohistochemical staining. The overall 5-year disease-specific survival rate is reported to be 81%, and the metastasis rate as 22%.^[4]^ To date, our patient has no recurrence, and we will continue follow-up as required.

## Author contributions

**Conceptualization:** Seok Soo Lee, Jang hoon Lee.

**Data curation:** Seok Soo Lee.

**Formal analysis:** Seok Soo Lee.

**Funding acquisition:** Seok Soo Lee, Jang hoon Lee.

**Investigation:** Seok Soo Lee.

**Methodology:** Seok Soo Lee.

**Project administration:** Seok Soo Lee.

**Resources:** Seok Soo Lee.

**Software:** Seok Soo Lee.

**Supervision:** Jang hoon Lee.

**Writing – original draft:** Seok Soo Lee.

**Writing – review & editing:** Seok Soo Lee.
